# Molecular Subtypes in Head and Neck Cancer Exhibit Distinct Patterns of Chromosomal Gain and Loss of Canonical Cancer Genes

**DOI:** 10.1371/journal.pone.0056823

**Published:** 2013-02-22

**Authors:** Vonn Walter, Xiaoying Yin, Matthew D. Wilkerson, Christopher R. Cabanski, Ni Zhao, Ying Du, Mei Kim Ang, Michele C. Hayward, Ashley H. Salazar, Katherine A. Hoadley, Karen Fritchie, Charles G. Sailey, Mark C. Weissler, William W. Shockley, Adam M. Zanation, Trevor Hackman, Leigh B. Thorne, William D. Funkhouser, Kenneth L. Muldrew, Andrew F. Olshan, Scott H. Randell, Fred A. Wright, Carol G. Shores, D. Neil Hayes

**Affiliations:** 1 Lineberger Comprehensive Cancer Center, University of North Carolina at Chapel Hill, Chapel Hill, North Carolina, United States of America; 2 Department of Otolaryngology, University of North Carolina at Chapel Hill, Chapel Hill, North Carolina, United States of America; 3 The Genome Institute, Washington University, St. Louis, Missouri, United States of America; 4 Department of Biostatistics, University of North Carolina at Chapel Hill, Chapel Hill, North Carolina, United States of America; 5 Department of Medical Oncology, National Cancer Centre Singapore, Singapore, Singapore; 6 Department of Pathology, Mayo Clinic, Rochester, Minnesota, United States of America; 7 Department of Pathology, University of Arkansas for Medical Sciences, Little Rock, Arkansas, United States of America; 8 Department of Pathology and Laboratory Medicine, University of North Carolina at Chapel Hill, Chapel Hill, North Carolina, United States of America; 9 Department of Pathology, University of Toledo, Toledo, Ohio, United States of America; 10 Department of Epidemiology, University of North Carolina at Chapel Hill, Chapel Hill, North Carolina, United States of America; 11 Department of Cell and Molecular Physiology, University of North Carolina at Chapel Hill, Chapel Hill, North Carolina, United States of America; 12 Department of Medicine, Division of Hematology and Oncology, University of North Carolina at Chapel Hill, Chapel Hill, United States of America; Barts & The London School of Medicine and Dentistry, Queen Mary University of London, United Kingdom

## Abstract

Head and neck squamous cell carcinoma (HNSCC) is a frequently fatal heterogeneous disease. Beyond the role of human papilloma virus (HPV), no validated molecular characterization of the disease has been established. Using an integrated genomic analysis and validation methodology we confirm four molecular classes of HNSCC (basal, mesenchymal, atypical, and classical) consistent with signatures established for squamous carcinoma of the lung, including deregulation of the KEAP1/NFE2L2 oxidative stress pathway, differential utilization of the lineage markers SOX2 and TP63, and preference for the oncogenes PIK3CA and EGFR. For potential clinical use the signatures are complimentary to classification by HPV infection status as well as the putative high risk marker CCND1 copy number gain. A molecular etiology for the subtypes is suggested by statistically significant chromosomal gains and losses and differential cell of origin expression patterns. Model systems representative of each of the four subtypes are also presented.

## Introduction

Head and neck squamous cell carcinoma (HNSCC) is a heterogeneous disease that represents the seventh most common form of cancer in the United States. Beyond the role of human papilloma virus (HPV), no validated molecular characterization of the disease has been established [1]–[4]. To further characterize the diversity of HNSCC as well as other tumors, our group and others have suggested gene expression (GE) subtypes as a means to prioritize the dominant genomic patterns within a specific tumor group [5]–[11]. Validated subtypes based primarily on GE profiling of breast cancer, glioblastoma, lung cancer, and others have garnered broad interest [5]–[7], [9]–[11]. Preliminary work has suggested that clinically relevant subtypes are also found in head and neck cancer [8], but the findings have not been replicated, no model systems have been proposed, and the etiology of the subtypes is obscure. In other tumor types the validation of molecular signatures has been established by the following approach: (i) the subtypes were shown to be statistically valid, (ii) genomic alterations underlying the subtypes were documented, and (iii) model systems representative of the expression subtypes were identified. The current study was conceived to address each of the points mentioned above. Because the goal of this study was to detect gene expression patterns and underlying genomic events that are present in HNSCC, the study design did not incorporate any molecular subtypes that were defined a priori – e.g. subtypes classified by HPV status.

## Results

### Unsupervised Discovery of HNSCC Expression Subtypes

**Figure 1:**
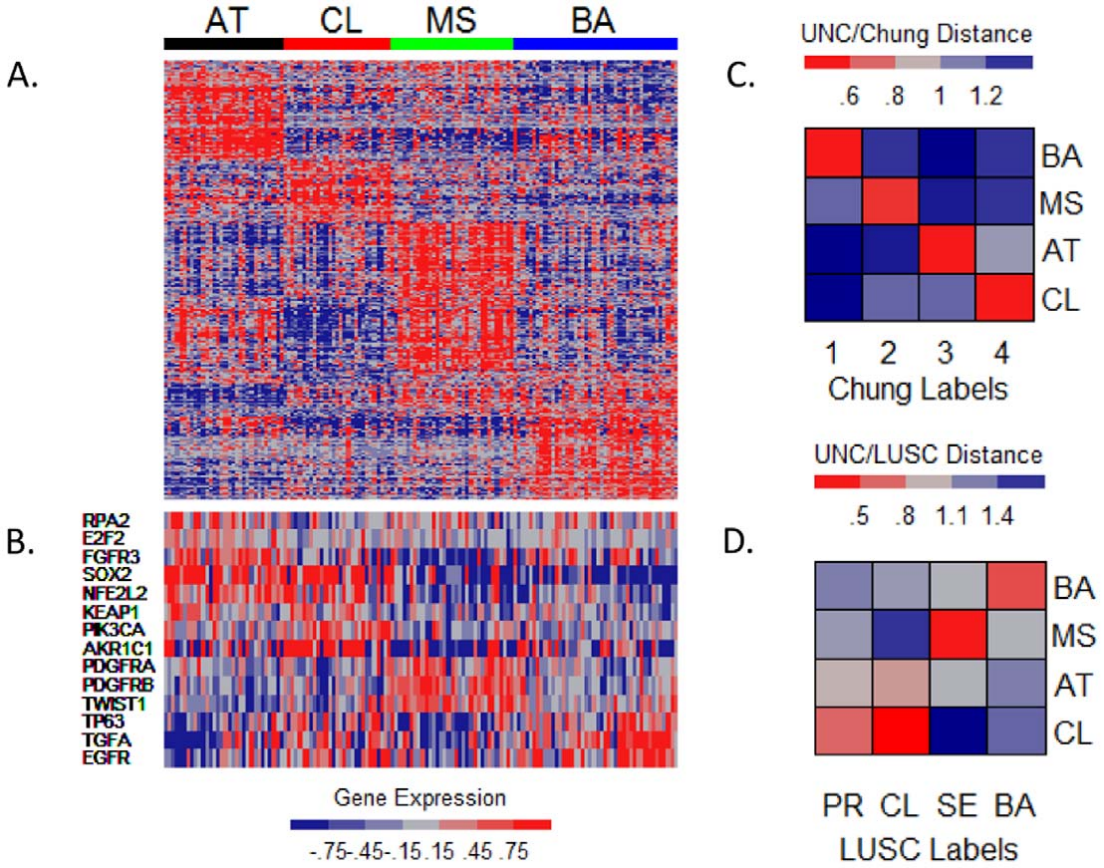
Gene Expression Subtypes in Head and Neck Squamous Cell Carcinoma. Heatmaps of the expression values of the 840 classifier genes (A) and select genes associated with HNSCC (B) for each of the expression subtypes. Validation heatmaps of the centroid-based distances between the centroids of the expression subtypes in the current study and those from Chung et al. (C) and the LUSC subtypes of Wilkerson et al. (D).

In order to address the question of whether statistically significant gene expression subtypes can be detected in HNSCC, we performed hierarchical clustering in an unsupervised and unbiased manner using well-established and objective techniques [7]. As in the prior work by Chung et al. [8], we documented the presence of four gene expression subtypes. Gene expression heatmaps (Figure 1A) and plots produced by ConsensusClusterPlus [12] (Figure S1 A – C) do not support the presence of additional statistically significant clusters in this dataset. A representative set of genes known or suspected to be relevant for head and neck cancer is shown in Figure 1B, and test statistics for the association of all genes in the dataset with tumor subtype are provided in Table S1. SigClust [13] analysis showed that the p-values for all of the pairwise comparisons of the expression subtypes were significant at the.05 level after applying a Bonferroni correction for multiple comparisons (Figure S1D). We refer to the expression subtypes as basal (BA), mesenchymal (MS), atypical (AT), and classical (CL) based on biological characteristics of genes highly expressed in each subtype.

### Clinical Characteristics

The clinical characteristics of the patients included in the current study represent a broad cross section of patients with HNSCC that is highly representative of the population seen in a typical clinical practice (Table 1). There was no correlation of tumor subtype with age, gender, race, alcohol use, pack years, or tumor size. Tumor subtypes were statistically associated with site, although all sites had tumors in each of the expression subtypes, with one exception (hypopharynx showed no BA). Additionally, no site contributed more than 58% of its samples to one expression subtype. No expression subtype was made up of more than 68% of tumors from a single site. Therefore, unlike other molecular markers such as HPV or p16, we conclude that expression subtypes captured a dimension of biology which was not limited to a single anatomic site [14]. There were additional statistically significant associations between tumor subtype and HPV status, treatment, node status, and overall stage. It is notable that more BA trended towards being well differentiated, whereas 13 of 16 poorly differentiated tumors were either MS or CL, although this difference was not statistically significant.

**Table 1:** Clinical Data.

	Total	Basal	Mesenchymal	Atypical	Classical	p-Value
Num. Patients	138	44	33	32	29	
Age (Years)						.75
Median	57	60	57	56.5	58	
Num. <40	9	5	3	1	0	
Sex						.64
Female	43	14	13	8	8	
Male	95	30	20	24	21	
Race						.34
Black	32	8	8	6	10	
White	104	36	24	26	18	
Alcohol Use						.44
None/Light	86	26	24	20	16	
Heavy	50	18	8	12	12	
Smoking						.11
Never/Light	27	13	6	6	2	
Current/Former	109	30	26	26	27	
Mean (Packyears)	36.0	36.7	33.1	30.1	45.0	.13
Differentiation						.10
Well	26	14	5	3	4	
Moderate	92	27	21	25	19	
Poor	19	3	7	3	6	
Tumor Site						1e-4*
Larynx	30	10	4	5	11	
Oral Cavity	55	30	18	2	5	
Oropharynx	34	3	5	20	6	
Hypopharynx	13	0	2	5	6	
Stage**						.034*
I	10	2	4	0	4	
II	14	8	1	2	3	
III	28	8	8	4	8	
IVa	77	26	16	22	13	
IVb	6	0	3	3	0	
IVc	10	0	0	1	0	
Tumor Status						.76
T0-T2	40	12	10	8	10	
T3-T4	77	30	16	16	15	
Node Status						.0026
N0-N1	66	30	14	6	16	
N2-N3	51	12	12	18	9	
Treatment						4.5e-6
Primary Chemo/RT	62	11	13	26	12	
Surgery	74	33	20	5	16	
HPV Status						.035
Negative	82	27	21	17	17	
Positive	14	1	3	8	2	
Chromosomal Instability Index	.056	.052	.048	.036	.136	2.2e-4

Summaries of select clinical covariates in the HNSCC expression subtypes. P-values for categorical variables were computed using Fisher’s Exact Test or a Monte Carlo version of Fisher’s Exact Test (p-values marked with *). P-values for continuous variables were computed using the Kruskal-Wallis test. **Stage I includes one patient that was classified as stage 0.

### Validation of Subtypes

We then turned our attention to the question of whether the expression subtypes detected in the current dataset corresponded to those previously reported by Chung et al. [8]. Wilkerson et al. [7] presented a method for comparing gene expression patterns found in expression subtypes across multiple studies. We use the same procedure, which is described more fully in the Methods section. Briefly, centroids of expression subtypes measure average gene expression values, and subtypes with concordant expression patterns produce centroids that are more highly correlated than subtypes with discordant expression patterns. A clear correspondence was observed (Figure 1C), with BA, MS, AT, and CL demonstrating the same expression patterns as the Chung subtypes 1, 2, 3, and 4, respectively. Having discovered four subtypes using independent and unbiased datasets and methods, we consider these four expression subtypes to be validated.

### Distinct Biological Processes and Similarities to Lung Squamous Cell Carcinoma

The expression patterns found in the subtypes suggest the presence of fundamental differences in the underlying biology of the associated tumors (Table S2). Gene expression in BA showed a strong similarity to the signature found in basal cells from the human airway epithelium, including high expression of genes such as *COL17A1,* which is associated with the extracellular matrix, the growth factor and receptor *TGFA* and *EGFR*, and the transcription factor *TP63* [14]. Tumors in MS were exemplified by elevated expression of genes associated with the epithelial-to-mesenchymal transition (EMT), including the mesenchymal markers *VIM* and *DES*, the transcription factor *TWIST1*, and the growth factor *HGF* [15], [16]. AT tumors had a strong HPV+ signature, as evidenced by elevated expression of *CDKN2A, LIG1*, and the transcription factor *RPA2* [17]. Tumors in CL, the subtype with the heaviest smoking history, showed high expression of genes associated with exposure to cigarette smoke, including the xenobiotic metabolism genes *AKR1C1/3* and *GPX2* [7], [18], [19] and the transcription factor *NFE2L2* [10].

Squamous cell carcinomas from different sites in the body share a number of molecular characteristics – e.g. loss of chromosome 3p and gain of chromosome 3q [20], [21] – so we hypothesized that a correspondence between our expression subtypes and recently reported lung squamous cell carcinoma (LUSC) expression subtypes [7] would be observed. To investigate a broader phenotype of squamous cell carcinomas of the upper aerodigestive tract, we extended the centroid predictor methodology and evaluated the correspondence of centroids from LUSC and HNSCC (Figure 1D). Remarkably, a clear pattern of correlation was observed in which the BA, MS, and CL subtypes of HNSCC corresponded to the LUSC basal, secretory, and classical subtypes, respectively, of Wilkerson et al. [7]. Examination of the TCGA LUSC data [10] provided additional compelling evidence of the underlying connections between the expression subtypes at the two tumor sites (Figure S2). The correspondence between the basal subtypes is notable because Wilkerson et al. [7] described time course experiments involving cultured human bronchial epithelial cells in which gene expression patterns at early time points showed a strong resemblance to those seen in the basal subtype of LUSC. Similarly, as shown in Table S3, we observed that the basal subtype of HNSCC is most similar to the day 3 time point in the time course data from the air liquid interface (ALI) model [22].

### DNA Copy Analysis by Subtype

**Figure 2:**
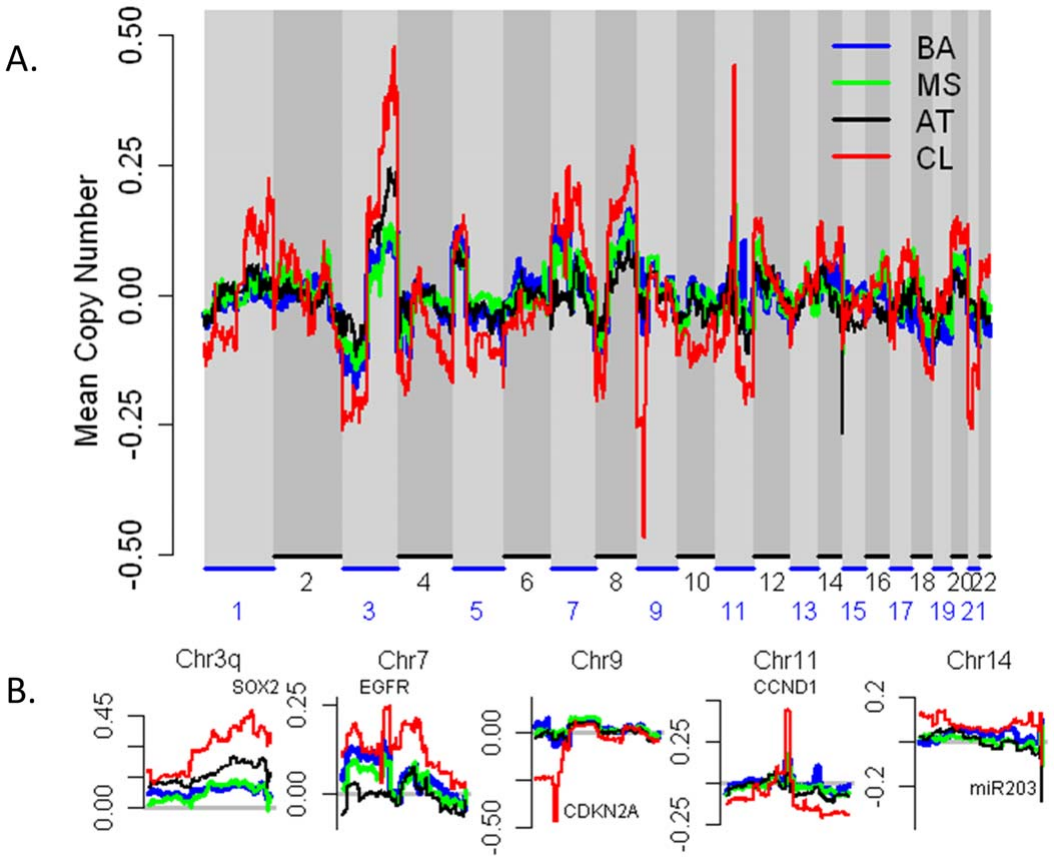
Copy Number Gains and Losses in the Expression Subtypes. Plots of the mean copy number values in the HNSCC expression subtypes after smoothing and outlier removal, both genome-wide (A) and for specific chromosomes or regions of interest (B).

We then turned our attention to the genomic alterations of HNSCC as measured by copy number (CN) arrays. First we confirmed many regions previously reported as altered in HNSCC, including gain of chromosomes 3q, 7p, and 11q (statistically significant gains are seen in both 11q13 and 11q22) and loss of chromosomes 3p, 9p, and 14q (Table S4 ). As has been seen in other tumors [11], there are both concordant and discordant patterns of copy number alteration in key regions of the genome as a function of tumor subtype (Figure 2, Table S5 ). For example, gains of 3q vary by expression subtype (p = .01), whereas no significant CN differences between the subtypes were detected in 11q13, which contains *CCND1* (p = 1). The canonical HNSCC 7p gain occurred in a region containing *EGFR*, but these alterations were found in BA, MS, and CL, not AT (p = .01). CN values in 3p were not significantly different across the subtypes (p = .47). Losses of the 9p region that contains *CDKN2A* were found in BA and CL only, and the CN differences were significant (p = .01). Focal CN loss was found in 14q32 for MS, CL, and is particularly pronounced in AT, but although this did not not reach statistical significance. This region contains miR203, which is notable because it targets ΔNp63 [23], one of six protein products of *TP63*. Chromosomal instability also varied considerably by subtype (p = 2.2e-4), as seen in Figure S3.

### Copy Number Changes and Differential Expression of Genes in Chromosome 3q by Expression Subtype

**Figure 3:**
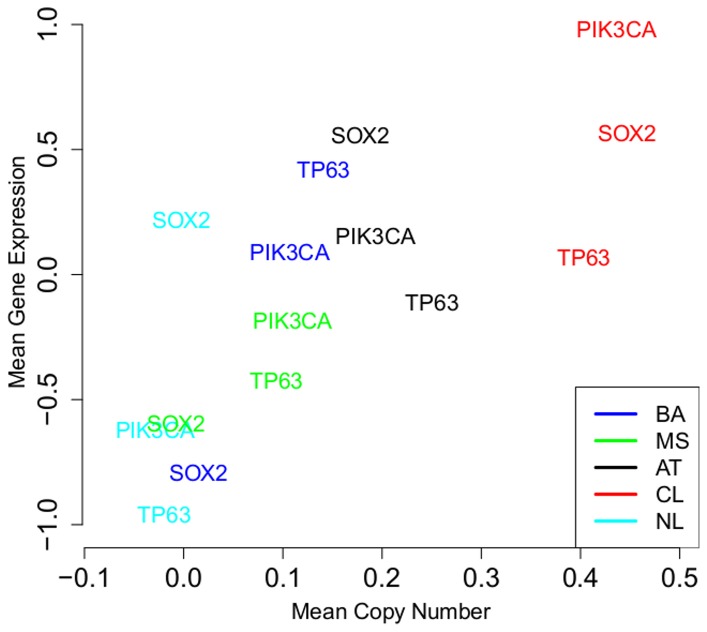
Average Gene Expression and Copy Number by Expression Subtype. Mean gene-specific copy number and gene expression values in the HNSCC expression subtypes and normal tonsil samples (NL) for genes in the 3q amplicon.

One of the quintessential genomic alterations associated with squamous cell carcinomas is gain of 3q [20], [21], and in the previous section we noted that the CN values in this region varied by expression subtype. Interestingly, there was a distinct differential proportional usage of the three genes typically discussed as the targets of the amplicon: *TP63, PIK3CA,* and *SOX2* (Figure 3). The CL and AT subtypes demonstrated proportionally higher expression of *SOX2* relative to MS and BA, which in fact appeared to express less *SOX2* than normal tonsil controls. By contrast, the BA subtype expressed dramatically higher levels of *TP63* than any other group. Similarly, although the MS subtype exhibited copy number gains in 3q, none of the putative target genes appeared to be expressed at levels higher than normal tonsil. Kruskal-Wallis tests showed that the expression of each of *TP63, PIK3CA,* and *SOX2* was associated with expression subtype after a Bonferroni adjustment for multiple testing (Table S6 ). This observation raises the possibility that the heterogeneity of HNSCC might in part be explained by differential usage of the transcription factors (*SOX2* and *TP63*) and oncogene (*PIK3CA*) in the 3q amplicon, which is more complex than has been previously reported [24]. It also suggests that differential usage of transcription factors and oncogenes, promoted in part by distinct copy number alterations, may contribute to the gene expression signatures that define the expression subtypes.

### Copy Number Events Involving Canonical Cancer Genes

Earlier we noted that the copy number values in gain and loss regions were associated with expression subtype. Now we describe similar findings that were obtained when gene-specific copy number values for genes known to play a role in HNSCC – *CCND1, CDKN2A,* and *EGFR* – were considered, not the broader regions discussed above. In the above discussion we stated that gains of 11q13 were not significantly different across the subtypes, and Table 2 shows that similar results were found when attention was restricted to gains of *CCND1*. In contrast, the frequency of *EGFR* gains ranged from 0% in AT to 31% in CL (p = .069), while the frequency of *CDKN2A* losses varied between 10% in MS to 63% in CL (p = .004). Both of these findings are concordant with the findings in the broader regions of 7p and 9p, respectively, described above.

**Table 2:** DNA Copy Number Events Involving Canonical Cancer Genes.

	Total	Basal	Mesenchymal	Atypical	Classical	p-Value
CCND1 Gain						.12
No	54	17	14	16	7	
Yes	30	9	7	4	10	
CDKN2A Loss						.004
No	63	20	19	18	6	
Yes	21	6	2	2	11	
Joint CCND1/CDKN2A Joint Event						.068
No	72	23	20	19	10	
Yes	12	3	1	1	7	
EGFR Gain						.069
No	72	22	18	20	12	
Yes	12	4	3	0	5	

Summaries of focal copy number events for specific genes in the HNSCC expression subtypes.

Past studies have detected associations between distinct genomic events, and these findings provided insight into either the underlying biology or the clinical management of cancer patients [25], [26]. In HNSCC, simultaneous *CCND1* gains and *CDKN2A* losses have been studied by Okami et al. [27] and Namazie et al. [28], with Namazie et al. detecting an association between these genomic events. We found that *CCND1* CN gains were associated with *CDNK2A* losses across all subtypes (Table S7), and that the joint event was associated with the expression subtypes (Table 2), thereby confirming and extending the results of Namazie et al.

### Clinical Outcomes by Expression Subtype and Focal Genomic Alterations

**Figure 4:**
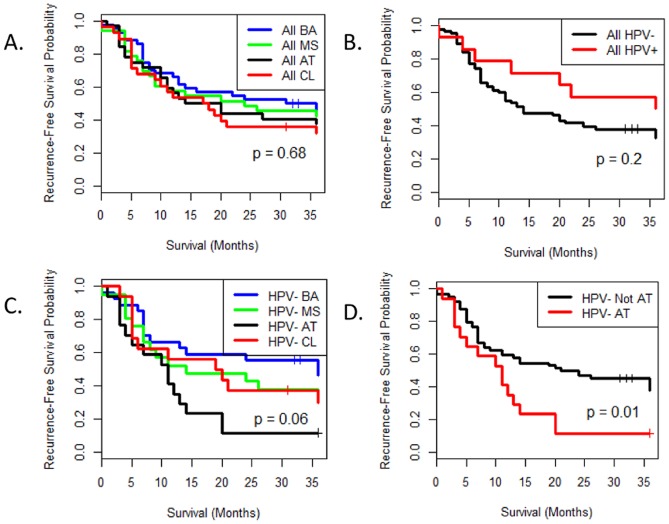
Recurrence-Free Survival in Expression Subtypes. Kaplan-Meier plots and Log-Rank Test p-values comparing recurrence-free survival times in all expression subtypes (A), HPV+ vs. HPV− subjects (B), all expression subtypes in HPV− subjects (C), and AT vs. non-AT in HPV− subjects (D). Statistical significance was assessed using the Log Rank Test.

Having parsed the set of nearly 140 HNSCC tumors into expression subtypes, and in light of known risk factors such as HPV, smoking, and alcohol use, we considered whether additional stratification for patient outcomes could be suggested. We first investigated whether the survival advantage reported by Chung et al. for “subtype 1″ could be reproduced in the current cohort. We were unable to confirm this result, and in the current study there was no association between recurrence-free survival and tumor subtype, either overall (Figure 4A) or when we restrict to late stage patients (not shown). These differences may be explained by the clinical heterogeneity of the disease combined with the fact that tumor site distributions in the two studies are markedly different.

In order to clarify whether known or suspected confounders might have affected our ability to detect subtype-specific differences in patient outcome, we evaluated the impact of HPV status on overall survival. We observed a relatively large but imprecise effect due to the overall small number of HPV+ patients (Figure 4B). We therefore considered it reasonable to re-evaluate the cohort with HPV+ patients excluded. Exclusion of HPV+ patients revealed that the AT subgroup demonstrated a particularly unfavorable outcome (Figure 4C), and this difference was statistically significant when compared to all other subtypes combined (Figure 4D). We then accessed an independent set of 122 tissue microarray (TMA) samples in an effort to validate this finding. Because array-based GE and immunohistochemistry (IHC) staining values are not comparable, it was not feasible to predict the tumor subtype of each TMA sample. Instead we used low EGFR and high p16 staining as a proxy for AT status. The difference in survival times was not statistically significant, but we obtained results similar to those described above (Figure S4).

We also investigated whether any focal copy number events were associated with clinical outcome. Previous studies have detected a correlation between CCND1 gains and decreased recurrence-free survival times in HNSCC [29]. We obtained similar findings when we examined the CN values for all tumor samples (Figure S5), although our results are marginally significant (p = .07). Remarkably few AT subjects exhibited *CCND1* gains (Table 2), and this suggests the presence of two largely distinct groups of patients with poor clinical outcomes: those with *CCND1* gains and those that are HPV− and AT. Figure S6 supports this conclusion.

### Expression Subtypes in Model Systems

The Cancer Cell Line Encyclopedia [30] contains genomic data from over 900 human cancer cell lines, including both GE and CN data from 19 esophageal and 18 upper aerodigestive tract cell lines. We applied our centroid predictor to the GE data from these cell lines and found that all four expression subtypes were present (Table S8). These findings are particularly compelling in light of the clinical relevance of the expression subtypes because they provide the basis for future studies involving model systems. Figure S7 provides examples to illustrate that subtype-specific CN events are also seen in the cell lines.

## Discussion

Our primary result was the detection of four gene expression subtypes in HNSCC – basal, mesenchymal, atypical, and classical. We also showed that these subtypes have biological and clinical relevance, and therefore they provide a useful and informative mechanism of classifying HNSCC tumors that complements existing methods based on histology and tumor site. Analysis of publicly available expression datasets revealed that these subtypes are reproducible in HNSCC [8] and are remarkably similar to those found in LUSC [7], [10]. Although gene expression patterns for the secretory LUSC subtype are similar to those seen in the mesenchymal subtype of HNSCC, we favor an alternate nomenclature. Data confirming the glandular origin of HNSCC is less compelling compared to that for the lung, and evidence of a mesenchymal signature is abundant [7]. While it would be possible to use the existing data to produce a gene predictor for HPV status, we did not attempt to do this because results of this nature were presented by Martinez et al. [31]. Regions of recurrent DNA copy number gain and loss were detected, some of which contain known oncogenes and tumor suppressors. The copy number values in certain aberrant regions were associated with tumor subtype, which suggests that copy number events may contribute to the development of expression subtypes. All of the expression subtypes were detected in HNSCC cell lines, a finding that provides the basis for future studies.

We now briefly discuss the definitions of the expression subtypes. Basal and classical were chosen because the expression patterns in these subtypes showed strong similarities to the basal and classical subtypes of LUSC. Wilkerson et al. compared the expression patterns in the LUSC subtypes to time course data from developing human bronchial epithelial cells, and they found that the basal subtype had similar expression patterns to those seen at early time points when basal cells are most common. Similarly, as shown in Table S2, we observed that the basal subtype of HNSCC is most similar to the day 3 time point in the time course data from the ALI model [22]. The classical subtype exhibits canonical genomic alterations associated with squamous cell carcinoma – e.g. deletion of 3p and 9p, amplification of 3q, and focal amplification of both *EGFR* and *CCND1*. Mesenchymal was selected based on pathway analysis indicative of an epithelial to mesenchymal transition. Finally, atypical was chosen because of the lack of either *EGFR* amplification or deletion of 9p.

The differences in the expression patterns found in the subtypes are clinically relevant. *TP63* produces six distinct proteins, and ΔNp63 is the most abundant isoform in HNSCC [32]. Yang et al. [33] show that ΔNp63 promotes cell proliferation. Chatterjee et al. [32] noted that exposure to cisplatin led to decreased levels of ΔNp63, so this treatment may be particularly effective for patients in BA. Barbieri et al. [34] showed that loss of *TP63* in HNSCC cell lines led to the acquisition of a mesenchymal phenotype, which is compelling in light of the low expression levels of *TP63* seen in MS. Martin and Cano [35] indicated that elevated expression of *TWIST1* or *BMI1* in HNSCC cell lines could increase the likelihood of invasiveness and migration. Because MS tumors exhibited an EMT phenotype and increased expression of both *TWIST1* and *BMI1*, these subjects may be more likely to develop distant metastases. The fact that *EGFR* is overexpressed in the vast majority of HNSCC tumors [36] makes *EGFR* inhibitors an attractive treatment option for this disease. However, these therapies are less likely to be effective in AT tumors because *EGFR* expression was lower than in the other expression subtypes. *SOX2* and *ALDH1* were highly expressed in AT and CL, and both of these genes are putative cancer stem cell markers because of their contributions to self-renewal and a pleuripotent phenotype [37], [38]. The protein product of *PIK3CA* is p110α, which phosphorylates AKT. Activated AKT contributes to the survival of tumor cells, and thus oncogenic transformation [39]. West et al. [40] showed that exposing normal lung epithelial cells to nicotine facilitates activation of AKT by making it dependent on PI3K alone. This observation, combined with the high levels of smoking seen in CL, suggests that PI3 kinase inhibitors provide an attractive treatment option for CL tumors.

There were several limitations to this study. First, we did not have GE, CN, and clinical data for all study subjects, which limited our ability to jointly analyze these variables. In addition, although the subtype labels were objectively defined by a clustering algorithm and the gene expression patterns were independently validated, the clinical associations were not. Copy number arrays were generated for all samples with sufficient quality and quantity of DNA. Unfortunately, over 20% of the arrays failed to meet standardized quality metrics. Also, it was not clear which isoform(s) of *TP63* were assayed by our gene expression arrays, and unfortunately the role that *TP63* plays in the basal subtype cannot be fully appreciated without knowledge of these isoforms. Because the HPV+ samples were removed when conducting our secondary survival analysis, these results should be viewed as exploratory and thus must be independently validated. Finally, the HPV status of all patients was not available.

In conclusion, we confirmed four molecular classes of HNSCC (basal, mesenchymal, atypical, and classical), consistent with signatures established for squamous carcinoma of the lung. Using an integrated genomic analysis and validation methodology, we documented subtypes identified by canonical tumor suppressor genes and oncogenes, including deregulation of the *KEAP1/NFE2L2* oxidative stress pathway, differential utilization of the lineage markers *SOX2* and *TP63*, and preference for the oncogenes *PIK3CA* and *EGFR*. For potential clinical use, the signatures are complimentary to classification by HPV infection status as well as the putative high risk marker *CCND1* copy number gain. A molecular etiology for the subtypes is suggested by statistically significant chromosomal gains and losses and differential cell of origin expression patterns. Model systems representative of each of the four subtypes were also presented.

## Materials and Methods

### Tumor Collection and Genetic Assays

After receiving written informed consent, frozen, surgically extracted, macrodissected head and neck tumors were collected at the University of North Carolina under Institutional Review Board protocol #01–1283. Tumor RNA was extracted and mRNA expression was assayed using Agilent 44K microarrays. Tumor DNA was extracted and DNA copy number was assayed using Affymetrix GenomeWide SNP 6.0 chips. A summary of all genetic data used in this study can be found in Table S9.

### mRNA Expression Analysis

Quality control procedures were applied to microarray probe-level intensity files. A total of 138 tumor arrays remained after removing low-quality arrays, duplicate arrays, and arrays from non-HNSCC samples. The normexp background correction and loess normalization procedures [41] were applied to the probe-level data. After log_2_ transformation, probes were matched to a common gene database to produce expression values for 15597 genes.

### Unsupervised Expression Subtype Discovery

The procedure described here is similar to that which appeared in Wilkerson et al. [7]. After expression values were gene median centered, gene variability was computed using the median absolution deviation. The 2500 most variable genes were selected. ConsensusClusterPlus [12] was used to perform unsupervised clustering for these genes in the 138 arrays. This procedure was performed with 1000 randomly selected sets of microarray samples using a sampling proportion of 80% and a distance metric equal to one minus the Pearson correlation coefficient.

### Statistical Significance of Gene Expression Patterns in Expression Subtypes

To confirm the statistical significance of four clusters, SigClust [13] was applied using the set of the 2500 most variable genes described above. All pairwise comparisons of the subtypes were examined using 1000 simulated samples and the original covariance estimation method.

### Differentially Expressed Genes and Metabolic Pathways

Differentially expressed genes were detected with the R package samr [42] using a median FDR threshold of.01. For each of the UNC subtypes we compared the gene expression values in the subtype to all other subtypes combined. DAVID [43] was then used to find KEGG pathways that showed enrichment for the highly expressed genes in each subtype. In addition, differentially expressed genes with known functional categories, e.g. transcription factors, were found by comparing the subtype-specific gene lists to known gene ontology categories [44].

### Published Expression Data

The microarray probe-level intensity files produced by Chung et al. [8] were subjected to background correction, normalization, and gene-level summarization procedures similar to those described above. This produced gene expression values for 60 subjects and 8224 genes. The subtype labels for these 60 arrays that appeared in [8] are referred to as Chung subtypes 1, 2, 3, and 4.

Summary RPKM values for 20,502 genes and 178 subjects were obtained based on the RNASeq data presented in [10]. The RPKM values were log_2_ transformed, and any gene that contained at least one missing value was removed from the analysis. This produced gene expression value for 15,314 genes.

### Validation of Expression Subtypes

Consensus clustering assigns a subtype label to every array. As a result, some arrays may not be representative of their subtype. Using silhouette widths [45], we identified a set of 125 “core” samples whose expression patterns were more similar to those of members of their own subtype than other subtypes. ClaNC [46], a classification method based on nearest centroids, was then applied to the UNC expression data from the core samples in an effort to create a set of classifier genes whose expression signature could be used to classify new samples. Minimizing the cross-validation error rate produced a list of 840 classifier genes (210 genes per subtype).

We identified the classifier genes whose expression values are also present in the Chung expression dataset, and then restricted the UNC and Chung expression datasets to these genes. After gene median centering each dataset separately, we found the centroid for each of the UNC and Chung subtypes by computing the median expression value for each gene over all arrays having the appropriate subtype label. As in [7], the distances between the UNC and Chung centroids were computed using a distance metric equal to one minus the Pearson correlation coefficient. This validation process was repeated using the LUSC data of Wilkerson et al. [7]. The RNASeq data from [10] was handled similarly with the following differences: (i) gene expression values from the UNC and log_2_(RPKM) values from the TCGA datasets were separately median centered and standardized by gene, (ii) predicted class labels were found, but class centroids were not computed.

### DNA Copy Number Analysis

CEL files were subjected to quality control procedures using the Affymetrix Genotyping Console, and arrays that produced contrast QC measurements above the default threshold of.4 were removed from subsequent analyses. The intensity values in the CEL files were then converted to log_2_ copy number values using the R package aroma [47] and a pooled collection of normal samples. A total of 107 arrays remained after manually reviewing the genome-wide copy number profiles, 84 of which have expression subtype labels. Missing values were imputed using the non-missing value from the closest probe. Segmentation was performed using DNAcopy [48].

Recurrent copy number gains and losses were detected with DiNAMIC [49] after smoothing and median centering the copy number profiles, as was done in [11]. DiNAMIC p-values were computed using 250 cyclic shifts, and gains and losses were classified as statistically significant if resulting p-values were less than.05. Regions harboring recurrent CN gains and losses were found using the bootstrap confidence interval procedure at level.95 with 500 bootstrap samples.

Associations between expression subtype and the five most significant gain and loss events were assessed as in [11]. First, for each subject the mean CN value over the corresponding confidence interval was computed. This was done with the smoothed and median centered CN values that were used to compute the confidence intervals, as described above. Then Kruskal-Wallis tests were applied to assess the association between each subject’s mean CN value and the expression subtype labels.

### Copy Number Gains and Losses of Canonical Cancer Genes

The gene-specific copy number was determined by computing the mean of all segmented copy number values at probes lying within or immediately adjacent to the gene. For each subject we classified a gene as having a copy number gain (loss) if the gene-specific copy number was above.35 (below −.35), which is approximately two standard deviations above (below) the mean of all segmented copy number values.

### Assessing HPV Status

Human papillomavirus was assessed using in situ hybridization. Slide deparaffinization, conditioning, and staining with INFORM HPV III Family 16 Probe (B; Ventana Medical Systems) were done on the Ventana Benchmark XT Autostainer according to the manufacturer’s protocol. The probes have affinities to HPV genotypes 16, 18, 31, 33, 35, 39, 45, 51, 52, 56, 58, and 66. Slides were scored as positive for HPV if a punctuate or diffuse pattern of signal were observed in the tumor nuclei.

### Statistical Analysis

R-2.12.2 and R-2.15.1 were used to perform all data analyses and create all figures. The statistical significance of associations between all categorical variables was assessed with Fisher’s Exact Test or a Monte Carlo version of Fisher’s Exact Test (p-values include an asterisk). Kruskal-Wallis tests were used to assess the statistical significance of associations of continuous variables with the expression subtypes. The survival package [50] was used to perform all survival analyses, and all p-values were computed using the log rank test. Recurrence-free survival (RFS) time was defined to be the time in months from tumor biopsy to death, recurrence, or loss to follow-up. Complete clinical data for all subjects, including RFS time, is presented in Table S10.

### Chromosomal Instability Index

For a given subject, we computed the median of the absolute value of the smoothed, segmented copy number values in each chromosome arm. The median of the arm-specific medians was defined to be the chromosomal instability index, which is similar to the definition that appears in [11].

### Cancer Cell Line Data

CN and GE data are available for 18 esophagus and 19 upper aerodigestive tract cell lines that were classified as squamous cell carcinoma in the Cancer Cell Line Encyclopedia [30]. GE data in the cell lines are available for 803 of the 840 genes in our classifier. After restricting to these common genes, we normalized the GE data for the cell lines so that it had the same gene-specific means and standard deviations as in our classifier. We then used the centroid-based method described above to predict expression subtypes for the cell lines.

### Data Availability

GE, CN, and select clinical data are available from GEO (accession number GSE39368).

## Supporting Information

Figure S1 **Evidence Supporting the Presence of Four Expression Subtypes.** Results are produced by ConsensusClusterPlus for 138 subjects and the 2500 most variable genes. (A) Heatmap of the consensus matrix for k  = 4 clusters. Entries in the consensus matrix measure the proportion of times two samples occur in the same cluster. High values (dark blue) show samples that are highly similar. (B) Plot of consensus cumulative distribution functions (CDFs) for different numbers of clusters k. Large differences between k = 2 (red), k = 3 (yellow), and k = 4 (green) shows greater stability with increasing numbers of clusters. Increasing k beyond 4 produces small gains. (C) The tracking plot shows that large numbers of samples change cluster label for k = 2, k = 3, and k = 4, indicating unstable clusters. However, only a small number of subjects change class between k = 4 and k = 5. (D) Bonferroni-adjusted SigClust p-values are highly significant (6 tests), indicating that all pairwise comparisons of the gene expression patterns in the four clusters are statistically significantly different.
(TIF)

Figure S2 **Expression Subtypes in HNSCC and LUSC.** Gene expression heatmap for the 715 of the 840 HNSCC from the current study (A) and the TCGA LUSC data (B). Strong similarities are seen between CL in both tumor types as well as MS in HNSCC and SE of LUSC. Gene expression heatmap for a representative set of genes known or suspected to be relevant for head and neck cancer from the core samples (C) and the TCGA LUSC data (D).
(TIF)

Figure S3 **Chromosomal Instability Index by Expression Subtype.** Boxplots of chromosomal instability indices in each of the gene expression subtypes as well as normal tonsil samples (NL).
(TIF)

Figure S4 **Kaplan-Meier Curves for HPV- Tissue Microarray Samples.** Kaplan-Meier curves illustrating differences in recurrence-free survival times for tissue microarray samples based on HPV status and immunohistochemical staining group (*EGFR* low/p16 high vs. others). Statistical significance was assessed using the log rank test.
(TIF)

Figure S5 **Kaplan-Meier Curves for CCND1 Copy Number Gains.** Kaplan-Meier curves illustrating differences in recurrence-free survival times for subjects with and without CCND1 copy number gains. Statistical significance was assessed using the log rank test.
(TIF)

Figure S6 **Kaplan-Meier Curves Illustrating Two Groups with Poor Survival Outcomes.** Kaplan-Meier curves illustrating differences in recurrence-free survival times for four mutually exclusive groups of patients: (1) HPV+ subjects (HPV+), (2) HPV− patients with CCND1 gains (CCND1 Gain), (3) HPV− patients without CCND1 gains that are AT (HPV− AT), (4) all remaining patients (Other). Statistical significance was assessed using the log rank test.
(TIF)

Figure S7 **Copy Number Plots from the Cancer Cell Line Encyclopedia Data.** Copy number plots show that genomic events detected in the UNC HNSCC cohort can also be found in the HNSCC cell lines from the Cancer Cell Line Encyclopedia. A. Amplifications in chromosome 3q are seen in all predicted subtypes, and the predicted classical subtype exhibits focal amplification of the region containing *SOX2*. B. SCC15 (predicted basal) exhibits focal amplification of *EGFR*, while HS840T (predicted atypical) has normal copy number. C. Both KYSE140 (predicted mesenchymal) and KYSE70 (predicted classical) exhibit focal deletion of *CDKN2A*. D. Both FADU (predicted mesenchymal) and SCC15 (predicted basal) exhibit focal amplification of CCND1. Note that gains of 11q22 are also seen for SCC15.
(TIF)

Table S1 **Differentially Expressed Genes in the Expression Subtypes.** The R package samr was used to identify genes that were differentially expressed when each subtype was compared to all other subtypes combined based on an FDR threshold of q = .01.
(XLSX)

Table S2 **Biological Characteristics of Expression Subtypes.** Table S1 lists genes that were differentially expressed when each subtype was compared to all other subtypes combined. Biological characteristics and molecular pathways representative of the highly expressed genes were then identified, as were other relevant genes (e.g. growth/transcription factors).
(DOCX)

Table S3 **Comparison of Expression Patterns in the Expression Subtypes and Time Course Data from the Air Liquid Interface Model.** Correlation-based distances between class centroids for the expression subtypes and time course centroids for the air liquid interface model show that the most similar expression subtype changes over time, with basal being the most similar at Day 3. Distance is equal to 1 minus the Pearson correlation coefficient of the centroids of interest.
(DOCX)

Table S4 **Regions Exhibiting Recurrent Copy Number Gain and Loss Events.** DiNAMIC was used to assess the statistical significance of recurrent copy number gain and loss events. Confidence intervals for the copy number events were also computed. For each event, “Marker” refers to the most significant copy number locus, while “Left” and “Right” refer to the boundaries of the associated confidence interval. Positions are hg18 genomic coordinates.
(XLSX)

Table S5 **Expression Subtypes Exhibit Different Copy Number Patterns in Regions of Chromosomal Gain and Loss.** Unadjusted Kruskal-Wallis Test p-values are given for associations between expression subtype and subject-specific mean copy numbers in the confidence intervals containing the five most significant gain and loss events. Adjusted p-values were computed using a Bonferroni adjustment (ten tests).
(DOCX)

Table S6 **Expression Subtypes Exhibit Different Expression Patterns of Oncogenes in Chromosome 3q.** Unadjusted Kruskal-Wallis Test p-values are given for associations between subject-specific expression of *TP63, PIK3CA,* and *SOX2* and expression subtype. Adjusted p-values were computed using a Bonferroni adjustment (three tests).
(DOCX)

Table S7 **Overall Association of** ***CCND1*** **Gains and** ***CDKN2A*** **Losses.** Two-by-two table illustrating *CCND1* gains and *CDKN2A* losses, together with Fisher’s Exact Test p-value.
(DOCX)

Table S8 **Predicted Expression Subtypes in Head and Neck Cancer Cell Lines.** Predicted gene expression subtypes in head and neck cancer samples of the Cancer Cell Line Encyclopedia obtained using the centroid predictor described in Methods.
(DOCX)

Table S9 **Summary of Datasets.** Summary of data and tissue types, sample sizes, and platforms for all datasets discussed herein.
(DOCX)

Table S10 **Clinical Data by Subject.** Clinical data for each of the 138 subjects that have expression subtypes.
(XLSX)
