# Correction: Molecular Subtypes in Head and Neck Cancer Exhibit Distinct Patterns of Chromosomal Gain and Loss of Canonical Cancer Genes

**DOI:** 10.1371/annotation/b42f61c5-cb7e-49ca-8cd6-6e1f7903ad08

**Published:** 2013-07-16

**Authors:** Vonn Walter, Xiaoying Yin, Matthew D. Wilkerson, Christopher R. Cabanski, Ni Zhao, Ying Du, Mei Kim Ang, Michele C. Hayward, Ashley H. Salazar, Katherine A. Hoadley, Karen Fritchie, Charles J. Sailey, Mark C. Weissler, William W. Shockley, Adam M. Zanation, Trevor Hackman, Leigh B. Thorne, William D. Funkhouser, Kenneth L. Muldrew, Andrew F. Olshan, Scott H. Randell, Fred A. Wright, Carol G. Shores, D. Neil Hayes

The name of the 12th author was given incorrectly. The correct name is: Charles J. Sailey. 

