# Correction: Molecular Subtypes in Head and Neck Cancer Exhibit Distinct Patterns of Chromosomal Gain and Loss of Canonical Cancer Genes

**DOI:** 10.1371/annotation/e9ad5950-a048-4a44-8e26-d8b07a9d4bb8

**Published:** 2014-01-16

**Authors:** Vonn Walter, Xiaoying Yin, Matthew D. Wilkerson, Christopher R. Cabanski, Ni Zhao, Ying Du, Mei Kim Ang, Michele C. Hayward, Ashley H. Salazar, Katherine A. Hoadley, Karen Fritchie, Charles G. Sailey, Mark C. Weissler, William W. Shockley, Adam M. Zanation, Trevor Hackman, Leigh B. Thorne, William D. Funkhouser, Kenneth L. Muldrew, Andrew F. Olshan, Scott H. Randell, Fred A. Wright, Carol G. Shores, D. Neil Hayes

In the supplementary materials, the 3rd cell line of Table S8 is incorrect, and should be listed as KYSE150_OESOPHAGUS. 

