# Correction: Molecular Subtypes in Head and Neck Cancer Exhibit Distinct Patterns of Chromosomal Gain and Loss of Canonical Cancer Genes

**DOI:** 10.1371/journal.pone.0194674

**Published:** 2018-03-15

**Authors:** Vonn Walter, Xiaoying Yin, Matthew D. Wilkerson, Christopher R. Cabanski, Ni Zhao, Ying Du, Mei Kim Ang, Michele C. Hayward, Ashley H. Salazar, Katherine A. Hoadley, Karen Fritchie, Charles J. Sailey, Mark C. Weissler, William W. Shockley, Adam M. Zanation, Trevor Hackman, Leigh B. Thorne, William D. Funkhouser, Kenneth L. Muldrew, Andrew F. Olshan, Scott H. Randell, Fred A. Wright, Carol G. Shores, D. Neil Hayes

In S7 Fig, the names of cell lines are incorrectly matched to the underlying data. Please see the corrected S7 Fig here.

## Supporting information

S7 Fig Copy number plots from the Cancer Cell Line Encyclopedia data. Copy number plots show that genomic events detected in the UNC HNSCC cohort can also be found in the HNSCC cell lines from the Cancer Cell Line Encyclopedia. A. Amplifications in chromosome 3q are seen in all predicted subtypes, and the predicted classical subtype exhibits focal amplification of the region containing *SOX2*. B. HSC2 (predicted basal) exhibits focal amplification of *EGFR*, while KYSE510 (predicted atypical) does not. C. Both BIRC31 (predicted mesenchymal) and KYSE180 (predicted classical) exhibit focal deletion of *CDKN2A*. D. Both SCC15 (predicted mesenchymal) and PECAPJ34 (predicted basal) exhibit focal amplification of CCND1. Note that gains of 11q22 are also seen for SCC15.
(TIF)
