# Supplementary figures and images for: Molecular Subtypes in Head and Neck Cancer Exhibit Distinct Patterns of Chromosomal Gain and Loss of Canonical Cancer Genes
Source: PLoS One. 2013 Feb 22;8(2):e56823. doi: 10.1371/journal.pone.0056823 (PMC3579892; doi:10.1371/journal.pone.0056823)

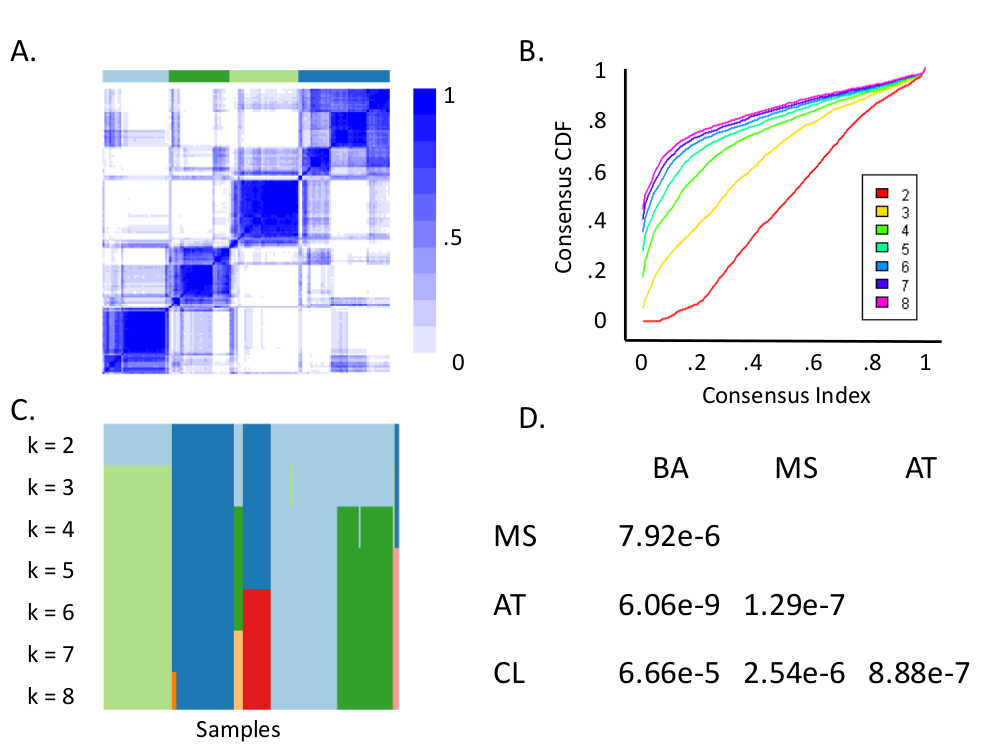

Supplement: Figure S1 — Evidence Supporting the Presence of Four Expression Subtypes. Results are produced by ConsensusClusterPlus for 138 subjects and the 2500 most variable genes. (A) Heatmap of the consensus matrix for k = 4 clusters. Entries in the consensus matrix measure the proportion of times two samples occur in the same cluster. High values (dark blue) show samples that are highly similar. (B) Plot of consensus cumulative distribution functions (CDFs) for different numbers of clusters k. Large differences between k = 2 (red), k = 3 (yellow), and k = 4 (green) shows greater stability with increasing numbers of clusters. Increasing k beyond 4 produces small gains. (C) The tracking plot shows that large numbers of samples change cluster label for k = 2, k = 3, and k = 4, indicating unstable clusters. However, only a small number of subjects change class between k = 4 and k = 5. (D) Bonferroni-adjusted SigClust p-values are highly significant (6 tests), indicating that all pairwise comparisons of the gene expression patterns in the four clusters are statistically significantly different. (TIF) [file pone.0056823.s001.tif]

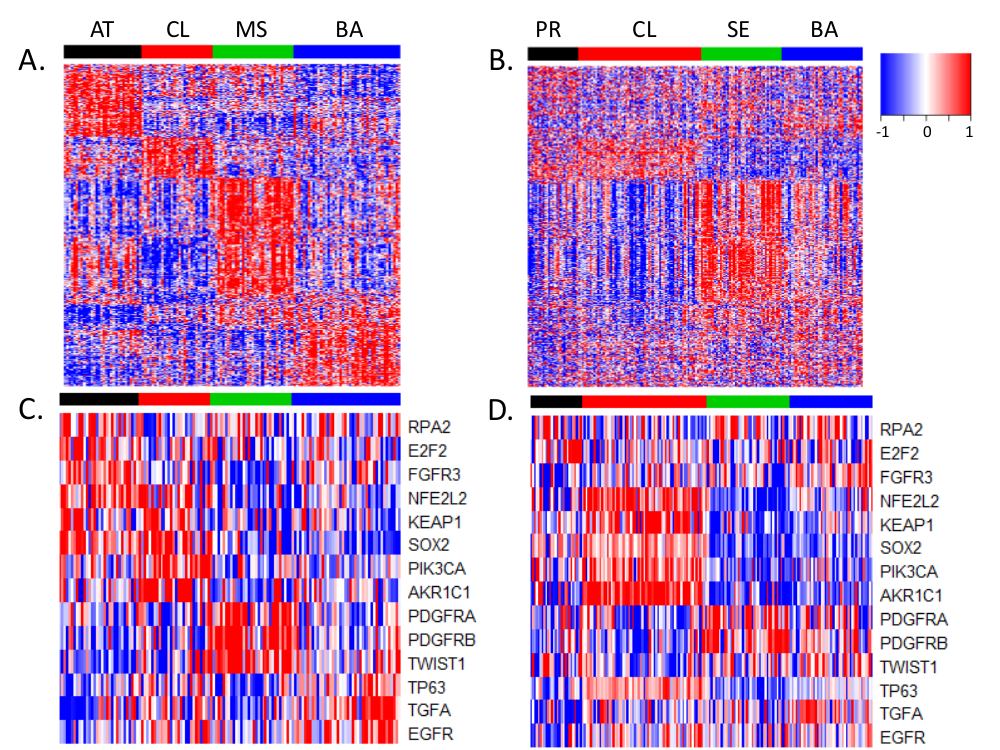

Supplement: Figure S2 — Expression Subtypes in HNSCC and LUSC. Gene expression heatmap for the 715 of the 840 HNSCC from the current study (A) and the TCGA LUSC data (B). Strong similarities are seen between CL in both tumor types as well as MS in HNSCC and SE of LUSC. Gene expression heatmap for a representative set of genes known or suspected to be relevant for head and neck cancer from the core samples (C) and the TCGA LUSC data (D). (TIF) [file pone.0056823.s002.tif]

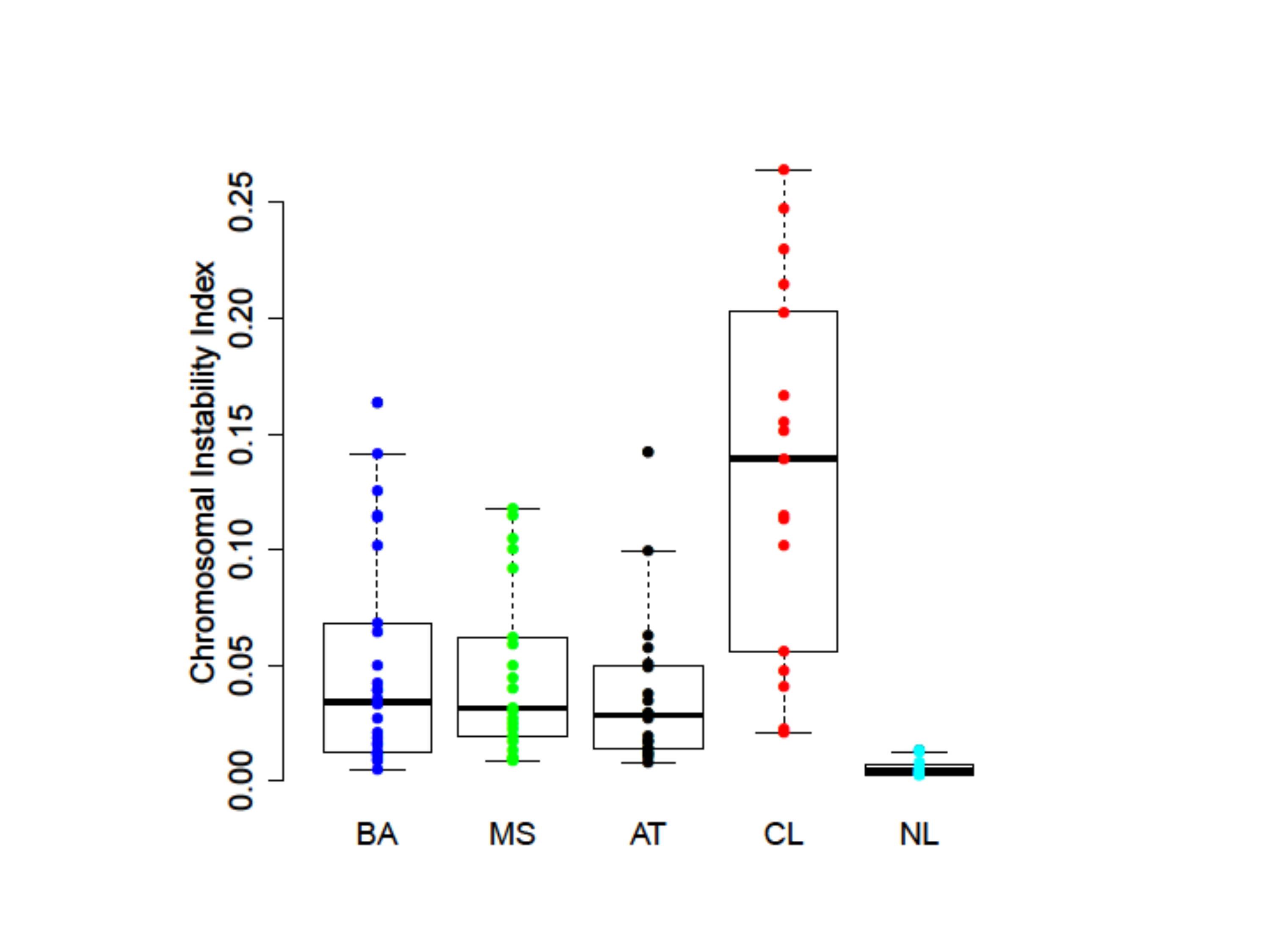

Supplement: Figure S3 — Chromosomal Instability Index by Expression Subtype. Boxplots of chromosomal instability indices in each of the gene expression subtypes as well as normal tonsil samples (NL). (TIF) [file pone.0056823.s003.tif]

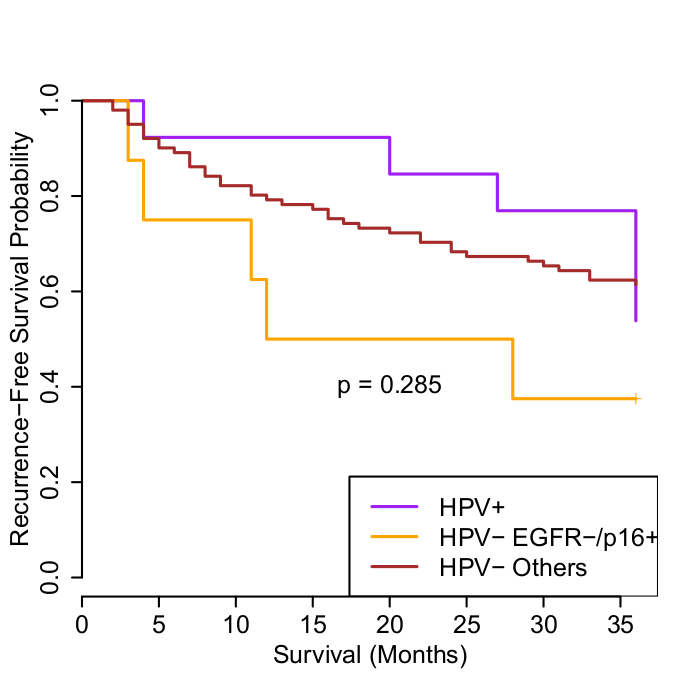

Supplement: Figure S4 — Kaplan-Meier Curves for HPV- Tissue Microarray Samples. Kaplan-Meier curves illustrating differences in recurrence-free survival times for tissue microarray samples based on HPV status and immunohistochemical staining group (EGFR low/p16 high vs. others). Statistical significance was assessed using the log rank test. (TIF) [file pone.0056823.s004.tif]

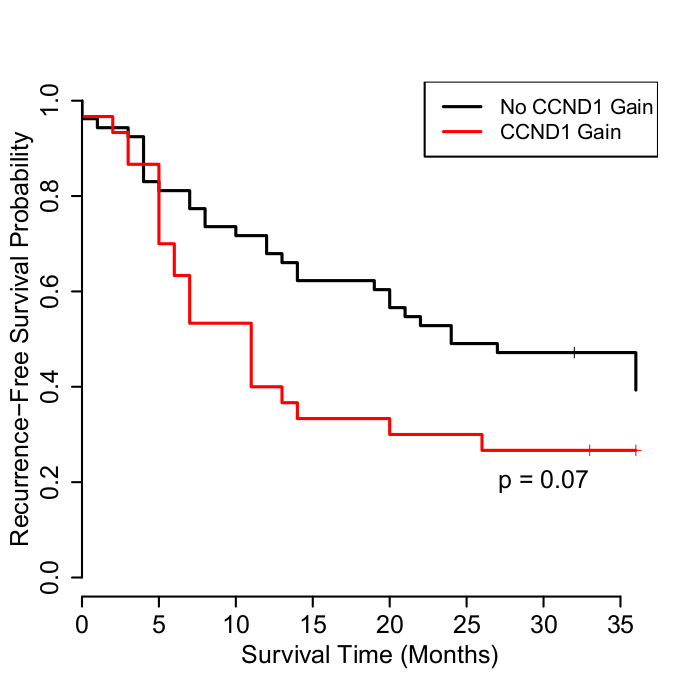

Supplement: Figure S5 — Kaplan-Meier Curves for CCND1 Copy Number Gains. Kaplan-Meier curves illustrating differences in recurrence-free survival times for subjects with and without CCND1 copy number gains. Statistical significance was assessed using the log rank test. (TIF) [file pone.0056823.s005.tif]

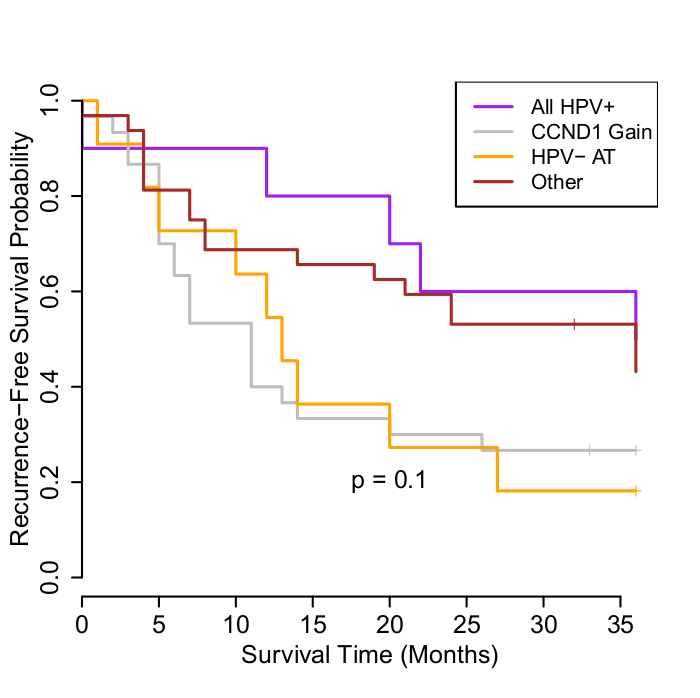

Supplement: Figure S6 — Kaplan-Meier Curves Illustrating Two Groups with Poor Survival Outcomes. Kaplan-Meier curves illustrating differences in recurrence-free survival times for four mutually exclusive groups of patients: (1) HPV+ subjects (HPV+), (2) HPV− patients with CCND1 gains (CCND1 Gain), (3) HPV− patients without CCND1 gains that are AT (HPV− AT), (4) all remaining patients (Other). Statistical significance was assessed using the log rank test. (TIF) [file pone.0056823.s006.tif]

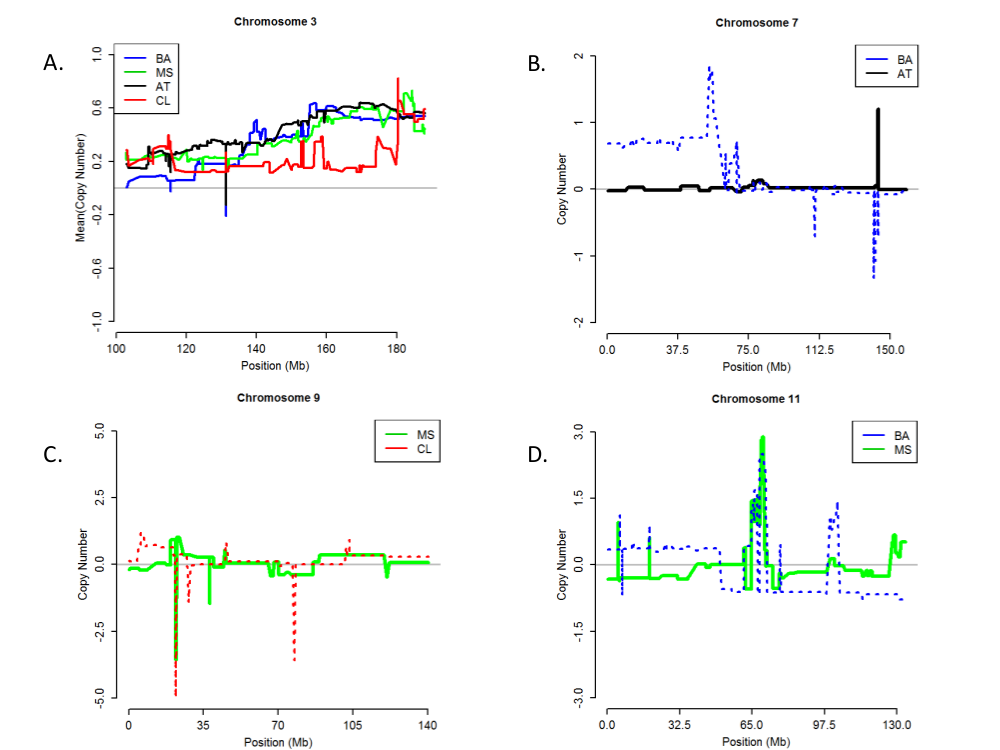

Supplement: Figure S7 — Copy Number Plots from the Cancer Cell Line Encyclopedia Data. Copy number plots show that genomic events detected in the UNC HNSCC cohort can also be found in the HNSCC cell lines from the Cancer Cell Line Encyclopedia. A. Amplifications in chromosome 3q are seen in all predicted subtypes, and the predicted classical subtype exhibits focal amplification of the region containing SOX2. B. SCC15 (predicted basal) exhibits focal amplification of EGFR, while HS840T (predicted atypical) has normal copy number. C. Both KYSE140 (predicted mesenchymal) and KYSE70 (predicted classical) exhibit focal deletion of CDKN2A. D. Both FADU (predicted mesenchymal) and SCC15 (predicted basal) exhibit focal amplification of CCND1. Note that gains of 11q22 are also seen for SCC15. (TIF) [file pone.0056823.s007.tif]
