# Supplementary material for: Molecular Subtypes in Head and Neck Cancer Exhibit Distinct Patterns of Chromosomal Gain and Loss of Canonical Cancer Genes
Source: PLoS One. 2013 Feb 22;8(2):e56823. doi: 10.1371/journal.pone.0056823 (PMC3579892; doi:10.1371/journal.pone.0056823)
Supplement: Table S2 — Biological Characteristics of Expression Subtypes. Table S1 lists genes that were differentially expressed when each subtype was compared to all other subtypes combined. Biological characteristics and molecular pathways representative of the highly expressed genes were then identified, as were other relevant genes (e.g. growth/transcription factors). (DOCX) [file pone.0056823.s009.docx]

| **Basal Subtype** | |
| --- | --- |
| Epidermal Development | *GJB5, COL17A1, S100A2, FABP5* |
| KEGG *Erbb* Signaling Pathway | *TGFA, EGFR, MAPK1, MAP2K1* |
| Growth/Transcription Factors | *E2F4, TP63* |
| **Mesenchymal Subtype** | |
| Epithelial-to-Mesenchymal Transition | *ACTA1, VIM, DES* |
| KEGG Focal Adhesion Pathway | *PDGFRA/B, COL1A2, COL3A1, LAMB2* |
| Growth/Transcription Factors | *HGF, TWIST1* |
| **Atypical Subtype** | |
| HPV Positivity | *CDKN2A, RPA2, LIG1* |
| KEGG Fatty Acid Metabolism Pathway | *ALDH3A1, ALDH9A1* |
| Growth/Transcription Factors | *E2F2, FGFR3* |
| **Classical Subtype** | |
| Xenobiotic Metabolism | *AKR1C1, AKR1C3, GPX2* |
| Transcription Factors | *NFE2L2, SOX2* |
| Cell Proliferation | *PIK3CA* |
