# Supplementary material for: Molecular Subtypes in Head and Neck Cancer Exhibit Distinct Patterns of Chromosomal Gain and Loss of Canonical Cancer Genes
Source: PLoS One. 2013 Feb 22;8(2):e56823. doi: 10.1371/journal.pone.0056823 (PMC3579892; doi:10.1371/journal.pone.0056823)
Supplement: Table S3 — Comparison of Expression Patterns in the Expression Subtypes and Time Course Data from the Air Liquid Interface Model. Correlation-based distances between class centroids for the expression subtypes and time course centroids for the air liquid interface model show that the most similar expression subtype changes over time, with basal being the most similar at Day 3. Distance is equal to 1 minus the Pearson correlation coefficient of the centroids of interest. (DOCX) [file pone.0056823.s010.docx]

|  | Baseline | Day 3 | Day 10 | Day 35 |
| --- | --- | --- | --- | --- |
| Basal | .85 | .79 | 1.15 | 1.23 |
| Mesenchymal | .67 | .95 | 1.26 | 1.08 |
| Atypical | 1.47 | 1.25 | .59 | .69 |
| Classical | .95 | .96 | 1.07 | .99 |
