# Supplementary material for: Molecular Subtypes in Head and Neck Cancer Exhibit Distinct Patterns of Chromosomal Gain and Loss of Canonical Cancer Genes
Source: PLoS One. 2013 Feb 22;8(2):e56823. doi: 10.1371/journal.pone.0056823 (PMC3579892; doi:10.1371/journal.pone.0056823)
Supplement: Table S5 — Expression Subtypes Exhibit Different Copy Number Patterns in Regions of Chromosomal Gain and Loss. Unadjusted Kruskal-Wallis Test p-values are given for associations between expression subtype and subject-specific mean copy numbers in the confidence intervals containing the five most significant gain and loss events. Adjusted p-values were computed using a Bonferroni adjustment (ten tests). (DOCX) [file pone.0056823.s012.docx]

| Gain | | | Loss | | |
| --- | --- | --- | --- | --- | --- |
| Chromosome | Unadjusted p-Value | Adjusted p-Value | Chromosome | Unadjusted p-Value | Adjusted p-Value |
| 11 | .072 | .72 | 14 | .10 | 1 |
| 3 | .001 | .01 | 3 | .047 | .47 |
| 8 | .014 | .14 | 9 | .001 | .01 |
| 5 | .72 | 1 | 8 | .26 | 1 |
| 7 | .001 | .01 | 5 | .065 | .65 |
