# Supplementary material for: Molecular Subtypes in Head and Neck Cancer Exhibit Distinct Patterns of Chromosomal Gain and Loss of Canonical Cancer Genes
Source: PLoS One. 2013 Feb 22;8(2):e56823. doi: 10.1371/journal.pone.0056823 (PMC3579892; doi:10.1371/journal.pone.0056823)
Supplement: Table S6 — Expression Subtypes Exhibit Different Expression Patterns of Oncogenes in Chromosome 3q. Unadjusted Kruskal-Wallis Test p-values are given for associations between subject-specific expression of TP63, PIK3CA, and SOX2 and expression subtype. Adjusted p-values were computed using a Bonferroni adjustment (three tests). (DOCX) [file pone.0056823.s013.docx]

| Gene | Basal | Mesenchymal | Atypical | Classical | Unadjusted p-Value | Adjusted p-Value |
| --- | --- | --- | --- | --- | --- | --- |
| TP63 | .42 | -.43 | -.11 | .067 | 1.4e-2 | 4.2e-2 |
| PIK3CA | .092 | -.18 | .15 | .98 | 5.4e-4 | 1.62e-3 |
| SOX2 | -.79 | -.59 | .56 | .57 | 1.4e-6 | 4.2e-6 |
