# Supplementary material for: Molecular Subtypes in Head and Neck Cancer Exhibit Distinct Patterns of Chromosomal Gain and Loss of Canonical Cancer Genes
Source: PLoS One. 2013 Feb 22;8(2):e56823. doi: 10.1371/journal.pone.0056823 (PMC3579892; doi:10.1371/journal.pone.0056823)
Supplement: Table S7 — Overall Association of CCND1 Gains and CDKN2A Losses. Two-by-two table illustrating CCND1 gains and CDKN2A losses, together with Fisher’s Exact Test p-value. (DOCX) [file pone.0056823.s014.docx]

|  | No CCND1 Gain | CCND1 Gain | Total | p-Value |
| --- | --- | --- | --- | --- |
| No CDKN2A Loss | 58 | 21 | 79 | .019 |
| CDKN2A Loss | 13 | 15 | 28 |  |
| Total | 71 | 36 | 107 |  |
