# Supplementary material for: Molecular Subtypes in Head and Neck Cancer Exhibit Distinct Patterns of Chromosomal Gain and Loss of Canonical Cancer Genes
Source: PLoS One. 2013 Feb 22;8(2):e56823. doi: 10.1371/journal.pone.0056823 (PMC3579892; doi:10.1371/journal.pone.0056823)
Supplement: Table S8 — Predicted Expression Subtypes in Head and Neck Cancer Cell Lines. Predicted gene expression subtypes in head and neck cancer samples of the Cancer Cell Line Encyclopedia obtained using the centroid predictor described in Methods. (DOCX) [file pone.0056823.s015.docx]

| Cell Line | Predicted Class |
| --- | --- |
| COLO680N_OESOPHAGUS | MS |
| KYSE140_OESOPHAGUS | CL |
| KYSE140_OESOPHAGUS | BA |
| KYSE180_OESOPHAGUS | CL |
| KYSE270_OESOPHAGUS | MS |
| KYSE30_OESOPHAGUS | AT |
| KYSE410_OESOPHAGUS | MS |
| KYSE450_OESOPHAGUS | CL |
| KYSE510_OESOPHAGUS | AT |
| KYSE520_OESOPHAGUS | MS |
| KYSE70_OESOPHAGUS | CL |
| OE19_OESOPHAGUS | AT |
| OE33_OESOPHAGUS | AT |
| TE11_OESOPHAGUS | CL |
| TE15_OESOPHAGUS | AT |
| TE1_OESOPHAGUS | MS |
| TE5_OESOPHAGUS | AT |
| TE9_OESOPHAGUS | AT |
| TT_OESOPHAGUS | CL |
| BICR31_UPPER_AERODIGESTIVE_TRACT | MS |
| CAL27_UPPER_AERODIGESTIVE_TRACT | BA |
| DETROIT562_UPPER_AERODIGESTIVE_TRACT | MS |
| FADU_UPPER_AERODIGESTIVE_TRACT | AT |
| HS840T_UPPER_AERODIGESTIVE_TRACT | MS |
| HSC2_UPPER_AERODIGESTIVE_TRACT | BA |
| HSC3_UPPER_AERODIGESTIVE_TRACT | BA |
| HSC4_UPPER_AERODIGESTIVE_TRACT | AT |
| PECAPJ15_UPPER_AERODIGESTIVE_TRACT | AT |
| PECAPJ34CLONEC12_UPPER_AERODIGESTIVE_TRACT | BA |
| PECAPJ41CLONED2_UPPER_AERODIGESTIVE_TRACT | MS |
| PECAPJ49_UPPER_AERODIGESTIVE_TRACT | MS |
| SCC15_UPPER_AERODIGESTIVE_TRACT | MS |
| SCC25_UPPER_AERODIGESTIVE_TRACT | MS |
| SCC4_UPPER_AERODIGESTIVE_TRACT | MS |
| SCC9_UPPER_AERODIGESTIVE_TRACT | BA |
| SNU1076_UPPER_AERODIGESTIVE_TRACT | AT |
| SNU899_UPPER_AERODIGESTIVE_TRACT | AT |
