# Supplementary material for: Molecular Subtypes in Head and Neck Cancer Exhibit Distinct Patterns of Chromosomal Gain and Loss of Canonical Cancer Genes
Source: PLoS One. 2013 Feb 22;8(2):e56823. doi: 10.1371/journal.pone.0056823 (PMC3579892; doi:10.1371/journal.pone.0056823)
Supplement: Table S9 — Summary of Datasets. Summary of data and tissue types, sample sizes, and platforms for all datasets discussed herein. (DOCX) [file pone.0056823.s016.docx]

| Dataset | Data Type | Tissue | N | Platform | Reference |
| --- | --- | --- | --- | --- | --- |
| UNC HNSCC | Gene Expression | HNSCC | 138^a^ | Agilent 4 x 44K | NA |
| UNC HNSCC | DNA Copy Number | HNSC | 107^a^ | Affymetrix GenomeWide SNP6.0 | NA |
| UNC TMA | Gene Expression | HNSCC | 122^b^ | Immuno- histochemistry | NA |
| Chung et al. | Gene Expression | HNSCC | 60^c^ | Agilent Human 1 cDNA | (8) |
| Wilkerson et al. | Gene Expression | LSCC | 56 | Agilent 4 x 44K | (7) |
| Cancer Cell Line Encyclopedia | Gene Expression | Esophageal and upper aerodigestive tract squamous cell carcinoma | 37 | Affymetrix Human Genome U133 Plus 2.0 | (30) |
| Cancer Cell Line Encyclopedia | DNA Copy Number | Esophageal and upper aerodigestive tract squamous cell carcinoma | 37 | Affymetrix GenomeWide SNP6.0 | (30) |
| TCGA Lung Squamous Cell Carcinoma | Gene Expression | LSCC | 178 | RNAseq | (10) |

1. 84 samples in UNC HNSCC have both gene expression and DNA copy number data.
2. No samples in UNC TMA belong to UNC HNSCC
3. 16 samples are common to UNC HNSCC and Chung et al.
