# Supplementary figures and images for: Correction: Molecular Subtypes in Head and Neck Cancer Exhibit Distinct Patterns of Chromosomal Gain and Loss of Canonical Cancer Genes
Source: PLoS One. 2018 Mar 15;13(3):e0194674. doi: 10.1371/journal.pone.0194674 (PMC5854434; doi:10.1371/journal.pone.0194674)

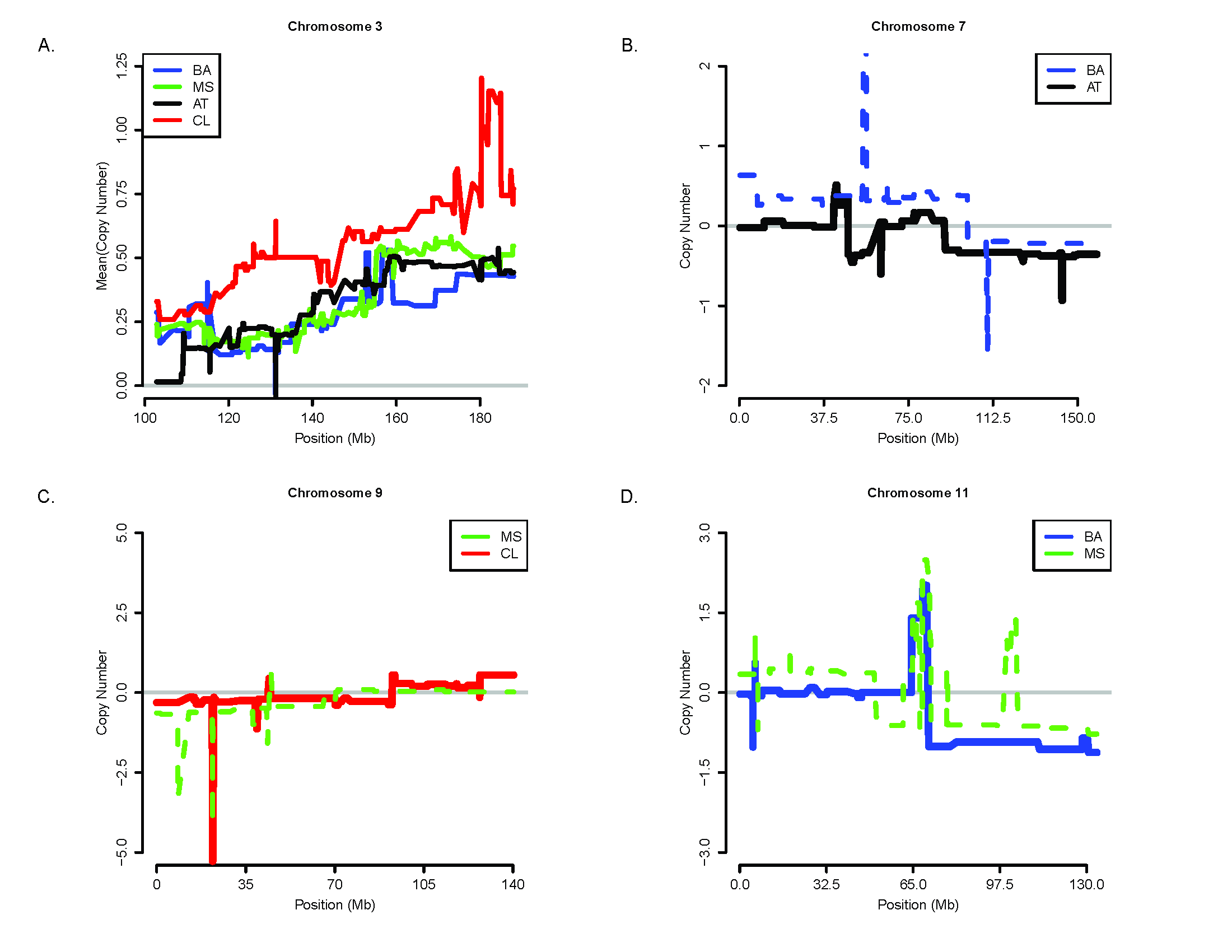

Supplement: S7 Fig — Copy number plots show that genomic events detected in the UNC HNSCC cohort can also be found in the HNSCC cell lines from the Cancer Cell Line Encyclopedia. A. Amplifications in chromosome 3q are seen in all predicted subtypes, and the predicted classical subtype exhibits focal amplification of the region containing SOX2. B. HSC2 (predicted basal) exhibits focal amplification of EGFR, while KYSE510 (predicted atypical) does not. C. Both BIRC31 (predicted mesenchymal) and KYSE180 (predicted classical) exhibit focal deletion of CDKN2A. D. Both SCC15 (predicted mesenchymal) and PECAPJ34 (predicted basal) exhibit focal amplification of CCND1. Note that gains of 11q22 are also seen for SCC15. (TIF) [file pone.0194674.s001.tif]
